# Inflammatory and Endothelial Dysfunction Biomarkers Predict Severe COVID-19 in Hospitalized Patients: Development of the CCBR Model

**DOI:** 10.3390/biomedicines14051074

**Published:** 2026-05-08

**Authors:** Sebastian Ciobanu, Aida-Isabela Adamescu, Cătălin Tilișcan, Andreea Mihaela Radu, Bogdan Popescu, Andrei Bogdan Văcărașu, Adrian Gabriel Marinescu, Victoria Aramă, Ștefan Sorin Aramă

**Affiliations:** 1 Faculty of Dental Medicine, Carol Davila University of Medicine and Pharmacy, 020021 Bucharest, Romania; 2 Emergency University Hospital, 050098 Bucharest, Romania; 3 National Institute of Infectious Disease “Prof. Dr. Matei Bals”, 020021 Bucharest, Romania; 4 University of Medicine and Pharmacy of Craiova, 200349 Craiova, Romania

**Keywords:** COVID-19 severity, biomarker-based prediction model, risk stratification, inflammatory biomarkers, endothelial dysfunction, neutrophil-to-lymphocyte ratio

## Abstract

**Background**: Early identification of patients at risk of severe COVID-19 is critical for timely interventions. We evaluated a biomarker-based risk stratification model, the Composite COVID-19 Biomarker Risk (CCBR) score, integrating age and inflammatory biomarkers (IL-6, PAI-1, LDH, neutrophil-to-lymphocyte ratio [NLR], and ferritin) measured at hospital admission to support early clinical risk assessment. **Methods**: In this retrospective single-center study, 235 hospitalized COVID-19 patients were classified into non-severe (*n* = 106) and severe (*n* = 129) groups. Biomarkers were measured within 24 h of admission. The CCBR score (0–6 points) was constructed by assigning one point to each parameter exceeding predefined cut-off values derived from published clinical thresholds and confirmed using receiver operating characteristic (ROC) curve analysis (Youden’s index). Patients were stratified into three risk categories: low risk (0–1 points), moderate risk (2–3 points), and high risk (4–6 points). Associations between CCBR scores and clinical outcomes, including severe disease, acute respiratory failure, dyspnea, complications, and antibiotic use, were assessed using logistic regression and ROC analyses. Internal validation was performed using split-sample validation and bootstrap resampling. **Results**: CCBR scores were significantly higher among patients with severe disease (*p* > 0.001). Each 1-point increase in CCBR was associated with a 6.78-fold increase in the odds of severe disease (OR = 6.78, *p* < 0.001). ROC analysis demonstrated moderate to good discriminative performance for severe disease (AUC = 0.714) and acute respiratory failure (AUC = 0.751). **Conclusions**: The CCBR score represents a simple biomarker-based model integrating inflammatory and endothelial dysfunction markers for early stratification of COVID-19 severity. This approach may assist clinicians in identifying patients at higher risk of severe disease and acute respiratory failure early during hospitalization.

## 1. Introduction

Although the global emergency phase of the COVID-19 pandemic has officially ended, ongoing scientific investigation remains essential. Continued viral evolution, particularly the emergence of new Omicron subvariants, has sustained transmission and increased reinfection rates [1]. Understanding the pathophysiological mechanisms underlying severe SARS-CoV-2 infection remains important for improving early clinical risk stratification and patient management.

Risk factors for severe COVID-19 can be broadly categorized as environmental, viral, or host-related. Among host factors, advanced age, male sex, and pre-existing comorbidities (e.g., arterial hypertension, diabetes mellitus, or malignancies) have consistently been associated with adverse outcomes [2]. Early identification of high-risk individuals enables timely clinical interventions, improves outcomes, and reduces preventable mortality [3].

Severe COVID-19 is characterized by a complex interplay between systemic inflammation, immune dysregulation, and endothelial dysfunction. These processes contribute to tissue injury, hypercoagulability, and organ dysfunction, which are hallmarks of critical disease. Several circulating biomarkers reflecting these mechanisms have therefore been investigated as potential predictors of disease severity [4].

Several biomarkers and composite indices, including inflammatory markers (e.g., ferritin, procalcitonin), nutritional indicators (e.g., albumin-based ratios), endothelial markers (e.g., Von Willebrand factor [vWF]), and cardiac injury markers (e.g., troponin), have been proposed to predict COVID-19 severity. In addition to individual biomarkers, composite indices integrating inflammatory, nutritional, and metabolic parameters, such as the C-reactive protein-to-albumin ratio (CAR), neutrophil-to-lymphocyte ratio (NLR), platelet-to-lymphocyte ratio (PLR), and other derived indices, have demonstrated significant prognostic value in COVID-19, including associations with disease severity, intensive care unit admission, and mortality [5]. These indices have been reported to achieve moderate to high discriminative performance in different cohorts, supporting their role as accessible and reproducible tools for risk stratification. Their indices are particularly attractive due to their availability and ability to reflect systemic inflammatory burden.

Furthermore, emerging evidence highlights the prognostic relevance of endothelial and coagulation biomarkers. Alterations in von Willebrand factor and its interaction with ADAMTS13 have been associated with disease severity and in-hospital mortality, reflecting COVID-19-associated endotheliopathy and thrombotic risk [6,7]. These findings support the concept that endothelial injury and dysregulated hemostasis are central mechanisms contributing to adverse clinical outcomes in SARS-CoV-2 infection.

Renal and metabolic markers have also gained attention as predictors of severity. Parameters such as urea levels and the urea-to-albumin ratio have been associated with mortality and reflect systemic catabolic state, hypoperfusion, and multi-organ dysfunction [8]. Such markers may provide complementary prognostic information beyond classical inflammatory parameters, particularly in critically ill populations. Additionally, novel biomarkers such as serum butyrylcholinesterase activity have been proposed as indicators of disease severity and mortality, reflecting both inflammatory and hepatic functional status [9].

In critically ill patients, the integration of serial biomarker measurements has been shown to improve prognostic accuracy, particularly in predicting outcomes such as extubation failure in COVID-19-associated acute respiratory distress syndrome [10]. This highlights the dynamic nature of biomarker expression and the potential added value of longitudinal assessment compared with single-time-point measurements.

Despite progress in clinical risk stratification, existing models often lack integration of key inflammatory and coagulation biomarkers. Biomarkers reflecting endothelial dysfunction and immunothrombosis may provide additional prognostic information, given the central role of vascular injury in severe COVID-19. Moreover, heterogeneity in biomarker selection and limited standardization across studies have hindered the development of widely applicable prognostic models. Furthermore, variability in study design and reported cut-off values limits direct comparability between studies. No consensus exists regarding an optimal model that integrates these pathophysiological pathways into a clinically applicable score.

To address this gap, we evaluated a biomarker-based model, referred to as the CCBR (composite COVID-19 biomarker risk) score, incorporating six variables: age, interleukin-6 (IL-6), plasminogen activator-1 (PAI-1), lactate dehydrogenase (LDH), neutrophil-to-lymphocyte ratio (NLR), and ferritin. These markers were selected based on their known pathophysiological relevance to COVID-19 and prior evidence supporting their individual prognostic value. While several scoring systems exist, few integrate inflammatory and coagulation biomarkers with demographic factors into a single, clinically practical model [5].

IL-6 is a central pro-inflammatory cytokine that plays a key role in cytokine storm syndromes, and it is often elevated in severe COVID-19 cases [11]. LDH reflects cellular damage and hypoxia and has been associated with intensive care unit (ICU) admission and prolonged hospitalization [12,13,14]. PAI-1 is a marker of endothelial dysfunction and impaired fibrinolysis and has been linked to thrombotic complications and increased mortality in COVID-19 [15]. Similarly, elevated NLR and ferritin levels correlate with hyperinflammation and adverse clinical outcomes [16,17,18,19]. These biomarkers capture key biological pathways involved in severe COVID-19, including hyperinflammation, endothelial dysfunction, and immunothrombosis, which play a central role in the progression to respiratory failure and systemic complications.

By integrating these parameters, the CCBR score aims to enable early and accurate risk stratification, guiding more personalized clinical management and resource allocation. The aim of this study was to evaluate the prognostic performance of the CCBR biomarker model for predicting severe disease and acute respiratory failure in hospitalized COVID-19 patients.

## 2. Materials and Methods

### 2.1. Study Design, Setting and Participants

This retrospective, observational, single-center study was conducted at the National Institute of Infectious Diseases “Prof. Dr. Matei Bals”, Bucharest, Romania, between March 2021 and October 2023. This study included patients aged ≥14 years with a confirmed SARS-CoV-2 infection, diagnosed using either real-time polymerase chain reaction (RT-PCR) or rapid antigen testing from nasopharyngeal or oropharyngeal swabs.

Exclusion criteria were incomplete data for key biomarkers (IL-6, PAI-1, LDH, NLR, ferritin), absence of a chest CT scan (computed tomography) at admission, pediatric patients (<14 years), and pregnant women. Information regarding prior SARS-CoV-2 infections was not systematically available.

### 2.2. Biomarker Measurement

Blood samples were collected within 24 h of admission. Serum IL-6, PAI-1, LDH, and ferritin levels were measured using standardized immunoassays and enzymatic colorimetric methods according to the manufacturer’s instructions.

Serum concentrations of inflammatory cytokines were determined using enzyme-linked immunosorbent assay (ELISA) techniques. IL-1 and IL-6 serum levels were quantified using commercially available Quantikine^®^ Immunoassay ELISA kits (catalogue number HS600B, R&D Systems, Minneapolis, MN, USA).

The neutrophil-to-lymphocyte ratio (NLR) was calculated from complete blood counts as the ratio of absolute neutrophil to lymphocyte counts.

All assays were performed following the manufacturers’ protocols, and results were interpreted according to the recommended calibration and quality-control procedures.

### 2.3. Composite Severity Score (CCBR Score)

The CCBR score was constructed by assigning one point to each of six parameters: age and five laboratory biomarkers (IL-6, PAI-1, LDH, NLR, and ferritin), resulting in a total score ranging from 0 to 6 points.

Cut-off values were selected based on previously published clinical thresholds and laboratory reference ranges and were further evaluated using ROC analysis to identify optimal discriminative values (Youden’s index).

Patients were stratified into three risk categories based on the CCBR score: low risk (0–1 points), moderate risk (2–3 points), and high risk (4–6 points).

Internal validation of the model was performed using a random split-sample approach. The dataset was randomly divided into a derivation cohort (70% of patients) and a validation cohort (30%). The predictive performance of the model was evaluated in both datasets using ROC analysis. Internal validation was further supported by using bootstrap resampling (1000 iterations) to assess the stability of the estimated coefficients and model performance.

Candidate variables were selected based on clinical relevance and previously reported associations with COVID-19 severity. Univariate analyses were initially performed to assess associations between individual variables and disease severity. Variables demonstrating statistical significance or strong clinical relevance were subsequently included in the multivariate logistic regression models.

To ensure independent contribution of each biomarker, multicollinearity was assessed using the variance inflation factors (VIF) and pairwise correlation coefficients. All VIF values were <2, indicating the absence of significant multicollinearity. Interaction terms between biomarkers were also tested in the logistic regression model and were not retained when non-significant, *p* > 0.05 (Table 1).

### 2.4. Exploratory Analysis of Additional Inflammatory Mediators

In addition to the laboratory parameters included in the CCBR score, exploratory analyses were performed for other inflammatory mediators that have been reported to be altered in SARS-CoV-2 infection and that were available in a subset of patients. These parameters were assessed in univariate analyses for potential correlations with disease severity, as well as with the CCBR score and its individual components. The aim of these analyses was to explore whether additional inflammatory markers might provide complementary prognostic information beyond the variables included in the CCBR score.

Although not all biomarkers demonstrated statistically significant associations with disease severity in the present cohort, IL-6 and PAI-1 were retained in the composite score because of their well-established biological role in the inflammatory and endothelial dysfunction pathways involved in severe COVID-19. Future studies with larger cohorts may further clarify their prognostic contribution.

Information regarding COVID-19 vaccination status at admission was retrieved from medical records when available.

### 2.5. Clinical Outcomes

The primary outcome was disease severity, classified according to World Health Organization (WHO) clinical criteria as mild, moderate, or severe (Table 2). Secondary outcomes included acute respiratory failure, in-hospital complications (cardiovascular, thromboembolic, neurological), bacterial superinfection, hepatic cytolysis, and in-hospital mortality.

### 2.6. Statistical Analysis

Continuous variables were expressed as mean ± standard deviation (SD) or median (interquartile range, IQR), depending on data distribution, and compared using Student’s *t*-Test or the Mann–Whitney U test, as appropriate. Categorical variables were reported as counts and percentages (%) and analyzed using the Chi-square or Fisher’s exact test, when appropriate.

To assess predictors of severe disease, univariate logistic regression analyses were initially performed. Variables demonstrating statistical significance or strong biological relevance were subsequently considered in the development of the composite score.

The predictive performance of the model was evaluated using binary logistic regression, with severe disease as the dependent variable. Odds ratios (Ors), 95% confidence intervals (CIs), and *p*-values were reported.

Model Discrimination was assessed using ROC analysis, and the area under the curve (AUC) was calculated to evaluate predictive performance. Model calibration was assessed using the Hosmer–Lemeshow goodness-of-fit test.

Internal validation of the model was performed using a split-sample approach. The dataset was randomly divided into a derivation cohort (70% of patients) and a validation cohort (30%). The predictive performance of the CCBR score was evaluated in both datasets using ROC analysis.

A *p*-value < 0.05 was considered statistically significant. All analyses were performed using SPSS v20 (IBM Group, Armonk, NY, USA).

Internal validation of the logistic regression model was further performed using a split-sample approach (70% derivation cohort and 30% validation cohort) and bootstrap resampling (1000 iterations) to assess the stability of the estimated coefficients and predictive performance.

### 2.7. Ethical Consideration

The study protocol was approved by the Institutional Ethics Committee of the National Institute of Infectious Diseases “Prof. Dr. Matei Bals (Approval No. C0408/2020, 20 October 2021). All procedures were conducted in accordance with the Declaration of Helsinki.

## 3. Results

### 3.1. Study Population

A total of 235 hospitalized patients were included, comprising 103 women (43.8%) and 132 men (56.2%). Patients were classified into two groups according to disease severity: non-severe disease (*n* = 106 patients, 45.1%) and severe disease (*n* = 129, 54.9%).

Patients were classified into two groups rather than into the three mild, moderate, and severe categories. This decision was based on the small number of patients with mild disease (*n* = 8), which would have limited statistical power and increased the risk of unstable estimates in subgroup analyses. Grouping into two categories ensured an adequate sample size per group for meaningful statistical comparisons and robust regression modeling.

The median age was significantly higher in patients with severe disease compared with those with non-severe disease (64 vs. 53 years, *p* = 0.025).

### 3.2. Comorbidities

Baseline comorbidities were generally similar across groups, except for arterial hypertension, which was significantly more prevalent in patients with severe disease (61.2% vs. 44.3%, *p* = 0.010). No significant differences were noted for obesity, smoking, diabetes (type 1 or 2), chronic kidney disease, COPD, malignancy, autoimmune conditions, or hepatic disease (*p* > 0.04) (Table 3). Arterial hypertension was defined based on documented medical history or ongoing antihypertensive treatment.

Vaccination status was available for a subset of patients, of whom 29 (12.3%) were vaccinated at the time of admission. Among vaccinated individuals, 4 patients (13.8%) developed severe disease, while 4 presented with moderate forms. Although the proportion of severe cases appeared lower among vaccinated individuals compared with the overall cohort, the small number of vaccinated patients limited the statistical power, and no robust conclusions could be drawn.

### 3.3. Biomarker Profiles and Composite Score (CCBR)

Biomarker analysis showed significantly higher levels of LDH, NLR, and ferritin in patients with severe disease compared with non-severe cases. No statistically significant differences were observed for IL-6 or PAI-1 in univariate comparisons.

In this multidimensional context, even variables with modest or non-significant standalone differences may enhance the overall predictive power when integrated with others. Prior studies have demonstrated that such markers, while sometimes variable across patient subsets, retain prognostic value within multivariate risk models (Table 4).

In addition to PAI-1, other coagulation-related markers commonly reported to be altered in COVID-19, including D-Dimer and fibrinogen, were measured in the study population. Exploratory analyses did not reveal consistent or statistically significant associations between these parameters and disease severity, patient outcomes, or the CCBR score. Furthermore, these markers did not demonstrate meaningful correlation with the individual components of the CCBR score and were therefore not included in the final model.

### 3.4. Univariate Logistic Regression for Severe vs. Non-Severe Disease

Univariate logistic regression was performed to evaluate the association between each clinically relevant variable and severe COVID-19 at admission. As shown in Table 5, age ≥ 65 years, LDH > 246 U/L, NLR > 5.25, ferritin ≥ 400 ng/mL, and hypertension were significantly associated with severe disease. In contrast, IL-6 ≥ 35 pg/mL, PAI-1 ≥ 300 pg/mL, diabetes mellitus, and sex were not significantly associated with severity in univariate analysis.

Despite their lack of statistical significance, IL-6 and PAI-1 were retained in the composite CCBR score due to their well-established biological roles in COVID-19 pathophysiology—IL-6 as a central mediator of hyperinflammation and PAI-1 as a regulator of fibrinolysis and thrombotic risk. Excluding these markers reduced the biological coherence and multidimensional scope of the score; therefore, they were preserved based on biological plausibility and prior literature.

### 3.5. Internal Validation Using Bootstrap Resampling

Internal validation of the logistic regression model was performed using bootstrap resampling with 1000 iterations to assess the stability of the estimated coefficients. The bootstrap analysis confirmed the robustness of the association between the CCBR score and severe COVID-19.

The bootstrapped odds ratio for severe disease was 6.64 (95% CI: 3.71–11.89, *p* < 0.001), which was consistent with the estimate obtained in the original logistic regression model. The narrow confidence interval and preservation of statistical significance across resampled datasets indicate good internal stability of the model.

These findings suggest that the predictive relationship between the CCBR score and disease severity was not driven by random variation within the sample, supporting the reliability of the model for early risk stratification in hospitalized COVID-19 patients.

The similarity between the bootstrapped estimates and the original regression coefficients suggests limited overfitting and adequate internal validity of the model.

Independent samples *t*-tests demonstrated that CCBR scores were significantly higher in patients with severe disease compared to non-severe cases (*p* < 0.001). Patients with severe COVID-19 had significantly higher CCBR scores compared with those with non-severe disease (median score 4 vs. 2, *p* < 0.001). This finding indicates that increasing CCBR values were strongly associated with greater disease severity.

For clinical interpretation, CCBR scores were categorized into three risk levels: low risk (0–1 points), moderate risk (2–3 points), and high risk (4–6 points). These cut-offs reflect increasing likelihood of severe disease or respiratory compromise and were derived from distributional analysis and clinical judgement.

Patients who developed clinical complications had higher CCBR scores compared with those without complications (median 4 vs. 2, *p* = 0.003). To formally assess this, an independent sample *t*-test was performed. Levene’s test for equality of variances was not significant, F (1, 209) = 0.138, *p* = 0.711), indicating that the assumption of homogeneity was met. The *t*-test revealed a statistically significant difference in severity scores between the two groups (t (209) = 2.99, *p* = 0.003), with a mean difference of 0.18 (95% CI (0.062, 0.305). The effect size (Cohen’s d = 0.41) indicated a small to moderate association. These results suggest that higher CCBR scores are associated with the presence of clinical complications during illness.

Consistent with these findings, patients who received antibiotic therapy also had higher CCBR scores compared to those who did not (M = 0.05 vs. −0.17), reflecting a moderate association (Cohen’s d = 0.49, *p* = 0.002). This suggests that patients with more severe clinical presentations, as captured by the composite severity score, were more likely to be treated with antibiotics.

Patients who developed acute respiratory failure had significantly higher CCBR scores than those who did not (median 4 vs. 2, *p* < 0.001). These findings reinforce the clinical utility of the CCBR score in identifying patients at risk for respiratory complications and systemic deterioration.

Arterial blood gas analysis was available for a subset of patients who developed acute respiratory failure. Exploratory correlation analyses between key blood gas parameters, including measures of oxygenation and carbon dioxide levels, and the CCBR score did not demonstrate statistically significant or clinically relevant associations. As such, these parameters were not included in further modeling.

By contrast, no significant difference in CCBR scores was observed between patients who developed hepatic cytolysis and those who did not (mean difference = −0.089, 95% CI, (−0.211, 0.0033), *p* = 0.152). Additionally, no significant difference was identified between men and women (mean difference = 0.11, 95% CI (−0.01, 0.23), *p* = 0.083), indicating that gender did not meaningfully influence disease severity in this cohort as measured by the composite score. Patients who died during the study were excluded from the main comparative analyses due to their extremely small number, which limited the statistical power and stability of estimates.

**Figure 1:**
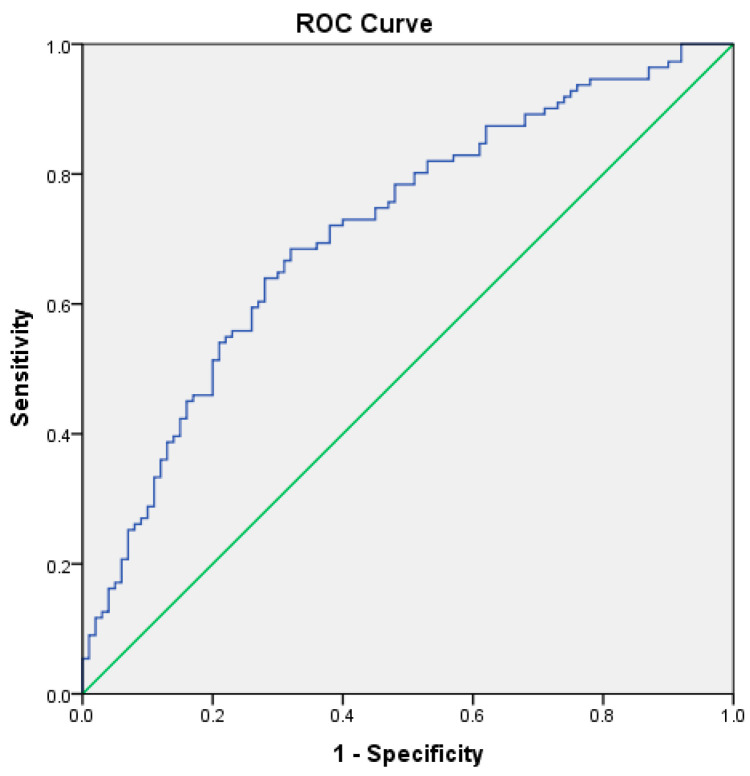
Predictive validity of severity score: ROC curve for severe forms of disease (AUC = 0.714).

**Figure 2:**
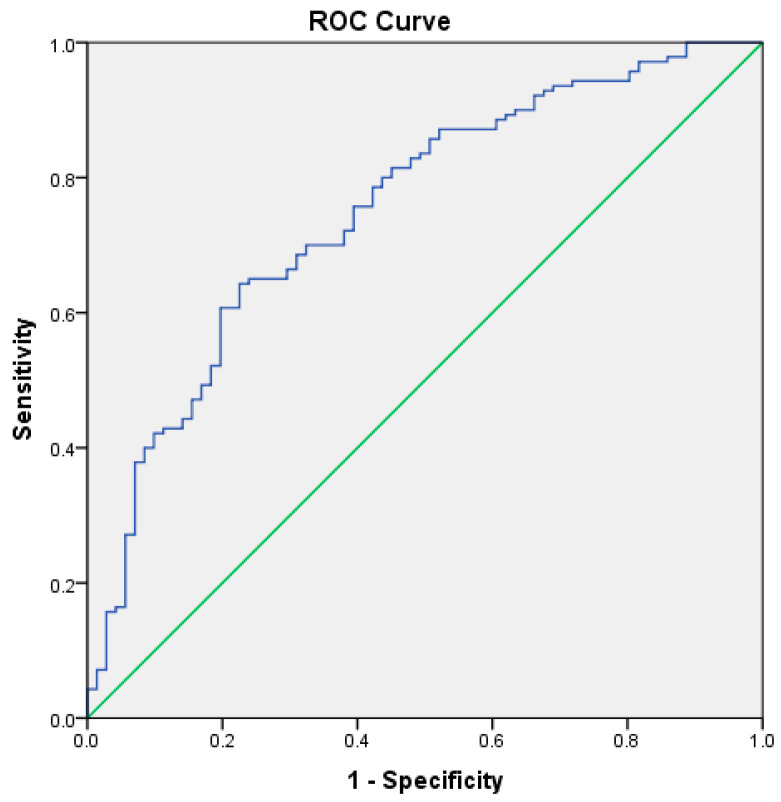
Predictive validity of severity score: ROC curve for the development of acute respiratory failure (AUC = 0.751).

**Figure 3:**
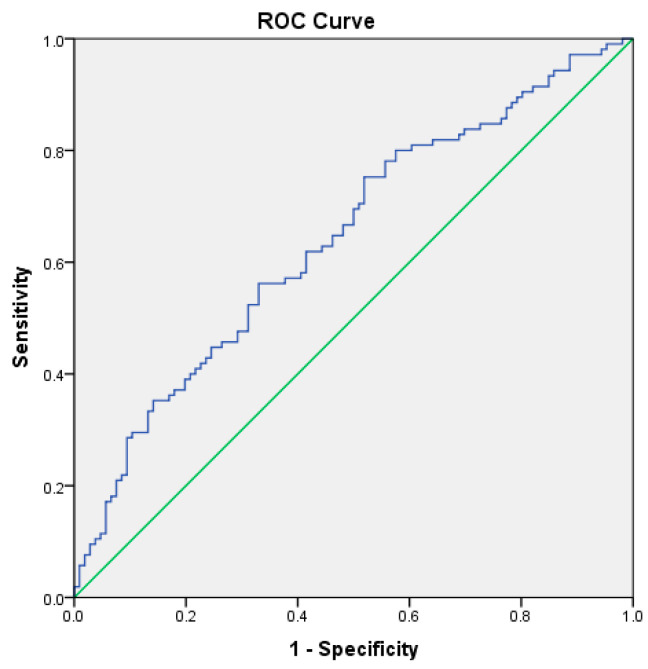
Predictive validity of severity score: ROC curve for the development of complications during hospitalization (AUC = 0.643).

ROC analysis demonstrated good discrimination for severe disease (AUC = 0.714, 95% CI: 0.645–0.783; *p* < 0.001), indicating good discriminatory ability. The score also performed well in predicting acute respiratory failure, with an AUC of 0. 751 (95% CI: 0.681–0.820; SE = 0.035, *p* < 0.001). Finally, the score demonstrated fair discrimination for predicting clinical complications, with an AUC of 0.643 (95% CI: 0.569–0.717; SE = 0.038, *p* < 0.001). The AUC values were significantly greater than 0.5, confirming that the CCBR score provided significant discriminative capacity for identifying patients at risk of severe disease and respiratory complications (Figure 1, Figure 2 and Figure 3).

We conducted logistic regression analyses to assess whether the composite severity score (CCBR) predicted the risk of severe vs. non-severe COVID-19. The model was deemed statistically significant based on the Omnibus Test of Model Coefficients (χ^2^ (1) = 30.35, *p* < 0.001), and the Hosmer–Lemeshow test confirmed good model fit (χ^2^ (8) = 3.53, *p* = 0.897). The model explained 13–18% of the variance in disease severity (Cox and Snell R^2^ = 0.134; Nagelkerke R^2^ = 0.179). Each 1-point increase in the CCBR score was associated with a 6.78-fold increase in the odds of severe COVID-19 (OR = 6.78, *p* < 0.001).

We also tested the ability of the CCBR score to predict acute respiratory failure (ARF). The model was statistically significant (Omnibus χ^2^ (1) = 37.41, *p* < 0.001) and accounted for 16–23% of the variance in ARF (Cox and Snell R^2^ = 0.162; Nagelkerke R^2^ = 0.225). The Hosmer–Lemeshow test indicated good model fit (χ^2^ (8) = 8.81, *p* = 0.359). Higher CCBR scores were significantly associated with an increased risk of ARF (B = −2.36, Exp(B) = 10.5, *p* < 0.001). The classification table showed that the model correctly identified 89.3% of patients who developed ARF, with an overall accuracy of 72%.

By contrast, the CCBR score was not a significant predictor of ICU admission in this cohort, likely due to the very few ICU admissions during the study period. Furthermore, these parameters did not demonstrate consistent associations with the individual components of the score. As such, they were not included in the final CCBR model.

## 4. Discussion

Severe COVID-19 is characterized by immune dysregulation involving excessive activation of the innate immune system together with impaired adaptive immune response. This imbalance promotes cytokine release, endothelial injury, and a pro-thrombotic state that contributes to organ dysfunction [20,21,22]. In addition to systemic inflammation, this hyperinflammatory state further amplifies endothelial injury and promotes a prothrombotic environment, thereby contributing to organ damage [23]. Elevated circulating levels of inflammatory cytokines, including IL-1, IL-6, IL-8, IL-10, and tumor necrosis factor-α, are commonly observed, with IL-6 showing the most pronounced increase. At the same time, patients with severe COVID-19 frequently exhibit reduced CD4 and CD8 T-cell values, reflecting compromised adaptive immunity and contributing to bilateral pneumonia, acute respiratory distress syndrome (ARDS), and multi-organ dysfunction [24].

IL-6 plays a central role in the inflammatory cascade of COVID-19 and has been widely associated with disease severity and adverse outcomes in several studies [25,26,27,28,29].

Lactate dehydrogenase (LDH) is a marker of cellular injury and tissue hypoxia. Elevated LDH levels have consistently been associated with severe COVID-19 and worse clinical outcomes, reflecting systemic inflammatory damage [30,31].

Ferritin, an acute-phase reactant, is frequently elevated in severe COVID-19 and reflects systemic inflammation and macrophage activation [32].

Compared with these markers, other widely studied biomarkers, including albumin-based indices (e.g., albumin ratio), renal function markers (e.g., urea or urea-to-albumin ratio), endothelial markers such as von Willebrand factor, and cardiac injury markers (e.g., troponin), have also demonstrated prognostic value in COVID-19. However, many of these markers reflect isolated aspects of disease pathophysiology or are more strongly associated with advanced or complicated stages of illness. In contrast, the CCBR score integrates biomarkers capturing inflammation, endothelial dysfunction, and tissue injury at an early stage, supporting its use for initial risk stratification [33,34,35].

In addition to the biomarkers included in the CCBR score, several other parameters have demonstrated prognostic value in COVID-19. Composite indices such as the CRP-to-albumin ratio and other inflammation-based scores have shown moderate to high discriminative performance across different populations. Endothelial biomarkers, particularly VWF and its interaction with ADATS13, have been associated with disease severity and mortality, reflecting the central role of endotheliopathy in COVID-19 [6,7]. Similarly, renal and metabolic markers, including urea and the urea-to-albumin ratio, have been linked to adverse outcomes and may reflect systemic catabolism and organ dysfunction [9]. Emerging biomarkers such as serum butyrylcholinesterase have also been proposed as predictors of severity and mortality [8].

However, many of these markers either reflect specific aspects of disease progression or are more strongly associated with advanced stages of illness. In contrast, the CCBR score integrates biomarkers capturing multiple pathophysiological pathways, including inflammation, endothelial dysfunction, and tissue injury, supporting its role as an early and multidimensional risk stratification tool.

Our composite CCBR score, integrating IL-6, PAI-1, LDH, NLR, ferritin, and age, aligns with this pathophysiological framework, providing a multidimensional approach to early risk stratification in hospitalized COVID-19 patients [36,37]. Importantly, this combination allows simultaneous assessment of inflammatory, endothelial, and immune-mediated mechanisms, distinguishing it from models based on single biomarkers or limited parameter sets. The CCBR should be regarded as an internally validated, proof-of-concept tool rather than a definitive prognostic model, and it requires external and prospective validation.

Although hypercoagulability is a recognized feature of COVID-19, exploratory analyses of additional coagulation markers, such as D-dimer and fibrinogen, did not provide incremental prognostic value in our cohort beyond the parameters included in the CCBR score. This finding may reflect the heterogeneity of coagulation abnormalities in SARS-CoV-2 infection and supports the selection of PAI-1 as a more integrative marker of fibrinolytic dysfunction within the proposed score. Larger prospective studies may further clarify the role of traditional coagulation markers in composite risk stratification models.

One of the most essential responsibilities in medical practice is the accurate stratification of risk and appropriate triage of patients. Scoring systems are among the most useful tools for this purpose. However, clear step-by-step guidance on how to develop prediction scores remains limited [38]. Severity scores classify patients according to illness intensity, assigning higher points as severity increases, whereas prognostic models extend this by predicting clinical outcomes using defined prognostic factors and mathematical factors [31]. However, the methodological heterogeneity and lack of standardized approaches for biomarker selection remain important limitations in the development of clinically applicable prognostic scores.

The CCBR score integrates six clinically relevant parameters reflecting inflammation, endothelial dysfunction, and tissue injury. Variables were selected based on biological relevance and statistical association with disease severity.

Validation analyses demonstrated that higher CCBR scores were significantly associated with severe disease, respiratory complications, and overall clinical deterioration. ROC analysis confirmed a moderate but clinically meaningful discriminative ability of the score.

### 4.1. Biological Rationale

The variables included in the CCBR score capture key pathophysiological mechanisms of COVID-19, including inflammation, endothelial dysfunction, immune dysregulation, and tissue injury [12]. LDH reflects tissue hypoxia and cell damage, and elevated NLR indicates systemic inflammation and neutrophil driven immune dysregulation [39]. In this study, LDH was used as a non-specific marker of tissue injury and hypoxia, without differentiation of isoforms. Similarly, ferritin was interpreted as an acute-phase reactant reflecting systemic inflammation rather than a marker of a specific cellular score [40]. PAI-1 levels represent thrombotic risk and endothelial dysfunction [35]. Age, a non-modifiable but highly predictive risk factor, is included due to its consistent association with severe outcomes. Together, these markers provide a multidimensional view of disease severity encompassing inflammation, immune response, coagulopathy, and host vulnerability.

### 4.2. Clinical Interpretation and Risk Stratification

Based on the distribution of scores in our cohort, 3 risk categories can be proposed. We propose the following risk categories:

- Low risk (0–1 points).
- Moderate risk (2–3 points).
- High risk (4–6 points).

These intervals provide a practical framework for applying the CCBR in real-time clinical settings, helping clinicians stratify patients efficiently. Although the scoring system supports stratification into three clinical risk categories, our cohort included only eight patients with mild disease, limiting the statistical power to analyze them as a separate group. As a result, we grouped patients into two broader categories—non-severe (mild and moderate) and severe—to enable more reliable comparisons and statistical analyses. Nonetheless, the score retains its capacity to differentiate between mild, moderate, and severe risk levels based on its biological rationale and distribution, offering a practical and scalable framework for application in diverse clinical settings.

Arterial blood gas abnormalities are a hallmark of acute respiratory failure in COVID-19, but the exploration analyses in our cohort did not identify a meaningful correlation between blood gas parameters and the CCBR score. This may reflect the fact that blood gas values are highly dynamic and influenced by disease stage, respiratory support, and therapeutic interventions, whereas the CCBR score aims to capture upstream systemic inflammatory and coagulation-related processes. These findings further support the complementary rather than overlapping nature of biochemical risk stratification and physiological respiratory assessment.

Although several inflammatory mediators have been implicated in the immunopathogenesis of COVID-19, exploratory analyses in our cohort did not identify additional markers that improved risk stratification beyond the parameters included in the CCBR score. This finding supports the robustness and clinical relevance of the selected variables, while also underscoring the complexity and heterogeneity of the inflammatory response in SARS-CoV-2 infection. Future studies in larger cohorts, with systematic measurement of cytokines and chemokines, may further clarify their role in composite prognostic models.

### 4.3. Comparison with the Existing Literature

Several prognostic tools have been proposed to predict COVID-19 severity, such as NEWS2, CALL, and the 4C mortality Score. These indices integrate various clinical and laboratory parameters and have shown good predictive value, with reported AUCs typically ranging between 0.70 and 0.88 in different populations [41,42,43].

In our cohort, the CCBR score achieved an AUC of 0.714 (95% CI:0.645–0.783) in predicting severe disease, which is comparable to those established scores, especially considering its simplicity and focus on biomarkers reflecting inflammation and coagulation [44]. These values are within the lower to intermediate range of previously reported models, which often demonstrate higher performance at the cost of increased complexity or reliance on non-routinely available parameters. However, most existing models focus on either clinical scores (e.g., NEWS-2) or single biomarkers [45], whereas the CCBR score integrates multiple biologically relevant biomarkers into a single composite model.

Unlike many existing models that rely on extensive clinical or imaging data, the CCBR score emphasizes biologically meaningful parameters measurable at admission, making it potentially useful in resource-limited settings. Nonetheless, direct head-to-head comparisons with these indices within the same dataset are warranted and will be pursued in future validation studies.

### 4.4. Strengths

This study has several key strengths:

- Multidimensional biomarker integration capturing immune-inflammatory and thrombotic mechanisms.
- Robust internal validation using ROC analysis and logistic regression.
- Clinical applicability, directly linking the score to important outcomes like acute respiratory failure, antibiotic use, and complications, making it actionable in real-world settings.
- A point-based format, enabling rapid bedside interpretation with widely available parameters.

### 4.5. Limitations

This study has several limitations that should be acknowledged. First, it was conducted at a single tertiary infectious diseases center, which may limit the generalizability of the findings to other healthcare settings or populations with different demographic and clinical characteristics. Second, the retrospective design induces potential selection bias and potential confounding, as data collection depends on the accuracy and completeness of medical records. Third, we exclude patients with missing biomarker data, which may have introduced bias if the missingness was not completely random. Additionally, not all potentially relevant biomarkers (e.g., troponin, vWF, albumin-based indices) were available in our dataset, which may have limited comparative evaluation with other proposed prognostic models.

To address these limitations, we are currently planning an external validation of the CCBR score in a multicentric cohort including both urban and regional hospitals. In addition, a prospective study is being designed to evaluate the score’s real-time applicability and to confirm its predictive performance across different patient populations. Despite these limitations, the CCBR score represents a practical, biomarker-based tool that can be readily tested and refined in future studies.

Physiological variables such as oxygenation and hemodynamic parameters, which may reflect disease severity, were not uniformly available and thus were not included in the multivariate analyses. The absence of this data may have led to residual confounding, and the findings should be interpreted in this context.

From an implementation perspective, the CCBR score relies largely on laboratory parameters that are routinely available in hospitalized patients. The main additional costs are related to IL-6 and PAI-1 testing, which are not universally performed in all healthcare systems. Although IL-6 and PAI-1 assays are not universally performed, they represent modest additional costs compared with standard laboratory testing and may be justified by improved early risk stratification.

Importantly, the CCBR score is intended for targeted use at hospital admission in patients at increased risk of severe disease, where early identification may support timely clinical decision-making, patient triage, and resource allocation. In this context, the potential benefits of improved risk stratification may outweigh the additional testing costs. Future studies should formally assess the cost-effectiveness of this approach in different healthcare settings.

Several biomarkers included in the CCBR score, such as LDH, ferritin, NLR, and PAI-1, may also be elevated in malignancies and chronic inflammatory diseases. While this could represent a potential confounding factor, the number of patients with such comorbidities in our cohort was small. Moreover, excluding these patients would have reduced statistical power and limited the real-world applicability of the score in hospitalized populations, where comorbid conditions are common. Future studies with larger cohorts may allow for stratified analyses or adjustment for these conditions.

Although chronic conditions such as arterial hypertension and diabetes mellitus were associated with disease severity in univariate analyses, they were not included in the final CCBR score. This decision was based on the lack of clear incremental prognostic value beyond age and the selected laboratory biomarkers, as well as the aim to maintain a parsimonious and clinically practical model. Furthermore, the CCBR score was designed to reflect acute inflammatory, coagulation, and tissue injury processes rather than baseline chronic conditions, which are already well-established risk factors for severe COVID-19. This approach enhances the score’s applicability for early risk stratification at hospital admission. While vaccination status was available, only a small proportion of patients were vaccinated (*n* = 29), limiting the ability to perform statistically meaningful comparisons. These findings should therefore be interpreted with caution.

### 4.6. Clinical Implications and Future Directions

Despite widespread vaccination and evolving viral variants, early identification of patients at risk for severe COVID-19 remains crucial. The CCBR score supports timely decision-making, resource allocation, and therapeutic prioritization (e.g., early corticosteroids or immunomodulators).

Future studies should focus on external and prospective validation, integration into EHR systems for automated scoring, application to other acute respiratory syndrome or viral infections and evaluation of long-term outcomes, including post-COVID sequelae.

## 5. Conclusions

The present study developed and internally validated the CCBR composite severity score, integrating age with biomarkers reflecting inflammation, endothelial dysfunction, and immune dysregulation to support early risk stratification in hospitalized COVID-19 severity. Each additional point in the score reflects a stepwise increase in disease severity, with values of 3 indicating moderate risk and 5–6 indicating high risk for severe outcomes, including acute respiratory failure.

The CCBR score demonstrated strong associations with clinically relevant endpoints such as severe disease, respiratory complications and in-hospital clinical deterioration. Logistic regression and ROC analyses confirmed a moderate but clinically meaningful predictive performance, supporting the value of this biomarker model as a simple and objective tool for early clinical risk assessment.

By integrating multiple biological pathways into a single composite index, the CCBR score provides a practical framework for identifying patients at increased risk of disease progression at hospital admission. This approach may support clinical decision-making, patient monitoring, and allocation of healthcare resources.

Although the model showed stable performance in internal validation analyses, external validation in larger, multicenter and prospective cohorts is required before routine clinical implementation. Future studies should also compare the CCBR score with existing prognostic indices to further define its clinical utility.

## Figures and Tables

**Table 1:** CCBR score components.

Variable	Cut-Off	Points Assigned	Rationale
Age	≥65	1	Advanced age is consistently associated with increased risk of COVID-19
IL-6	>35 pg/mL	1	Elevated IL-6 reflects systemic inflammation and cytokine activation
PAI-1	≥300 ng/mL	1	Marker of endothelial dysfunction and impaired fibrinolysis, associated with thrombotic complications
LDH	>246 U/L	1	Marker of tissue injury and hypoxia, associated with severe disease and ICU admission
NLR	>5.25	1	Elevated neutrophil-to-lymphocyte ratio reflects systemic inflammatory response
Ferritin	≥400 ng/mL	1	Elevated ferritin reflects hyperinflammation and dysregulated immune response
Total score 0–6			Higher scores indicate increased risk of severe COVID-19

Cut-off values were selected based on previously published clinical thresholds and confirmed using receiver operating characteristic (ROC) curve analysis. Abbreviations: IL-6, interleukin 6; PAI-1, plasminogen activator inhibitor-1; LDH, lactate dehydrogenase; NLR, neutrophil-to-lymphocyte ratio.

**Table 2:** Classification of illness severity based on symptoms, oxygen saturation, and imaging findings.

	Symptoms (e.g., Fever, Cough, Sore Throat, Myalgias, Nausea, Vomiting)	Peripheral Oxygen Saturation	Imaging Findings
Mild Moderate	Present	>94%	NO pulmonary abnormalities
	Present	>94%	Mild pulmonary abnormalities
Severe	Present	<94%	Pulmonary infiltrates > 50% affecting of the lung fields

**Table 3:** Distribution of baseline comorbidities by COVID-19 disease severity.

	Non-Severe Forms of Disease N (%)	Severe Forms of Disease N (%)	*p*-Value
Obesity	40 (45.3)	60 (46.5)	0.643
Smoker	3 (2.8)	4 (3.1)	0.656
aHTN	47 (44.3)	79 (61.2)	0.010
T2DM	16 (15.1)	14 (10.9)	0.332
T1DM	6 (5.7)	7 (5.4)	0.938
COPD	2 (1.9)	1 (0.8)	0.450
CKD	7 (6.6)	9 (7)	0.910
Dialysis	0	1 (0.8)	0.364
Hematological disease	1 (0.9)	3 (2.3)	0.415
Neoplasia	7 (6.2)	10 (7.8)	0.812
Chronic hepatic disease	11 (10.4)	13 (10.1)	0.940
Autoimmune disease	4 (3.8)	4 (3.1)	0.807
Other chronic conditions	74 (69.8)	97 (75.2)	0.519

Note: data are expressed as N (%). Abbreviations: aHTN, arterial hypertension; T2DM, non-insulin-dependent (type II) diabetes mellitus; T1DM, insulin-dependent (type I) diabetes mellitus; COPD, chronic obstructive pulmonary disease; CKD, chronic kidney disease.

**Table 4:** Median biomarker values in non-severe vs. severe COVID-19 cases.

	Median Value in Non-Severe Cases	Median Value in Severe Cases	*p*-Value
Age (years)	53	64	0.025
IL-6 (pg/mL)	122	134	0.664
PAI-1 (ng/mL)	314	297	0.466
LDH	270	375	0.026
NLR	4	7	0.000
Ferritin (ng/mL)	355	513	0.014

Abbreviations: IL-6, interleukin 6; PAI-1, plasminogen activator inhibitor-1; LDH, lactate dehydrogenase; NLR, neutrophil-to-lymphocyte ratio.

**Table 5:** Univariate logistic regression for severe vs. non-severe COVID-19 at admission.

Variable	OR	95% CI	*p*-Value
Age ≥ 65 years	2.12	1.35–3.33	0.001
LDH > 246 U/L	2.85	1.70–4.77	<0.001
NLR > 5.25	3.21	1.95–5.29	<0.001
Ferritin ≥ 400 ng/mL	1.74	1.12–2.72	0.015
Arterial hypertension	1.97	1.19–3.25	0.008
IL-6 ≥ 35 pg/mL	1.22	0.76–1.96	0.40
PAI-1 ≥ 300 ng/mL	1.11	0.71–1.73	0.63
Diabetes mellitus	0.78	0.41–1.48	0.45

Abbreviations: LDH, lactate dehydrogenase; IL-6, interleukin 6; PAI-1, plasminogen activator inhibitor-1; NLR, neutrophil-to-lymphocyte ratio.

## Data Availability

The original contributions presented in this study are included in the article. Further inquiries can be directed to the corresponding authors.
